# Novel Dual AChE and ROCK2 Inhibitor Induces Neurogenesis via PTEN/AKT Pathway in Alzheimer’s Disease Model

**DOI:** 10.3390/ijms232314788

**Published:** 2022-11-26

**Authors:** Natália Chermont dos Santos Moreira, Elvira Regina Tamarozzi, Jessica Ellen Barbosa de Freitas Lima, Larissa de Oliveira Piassi, Ivone Carvalho, Geraldo Aleixo Passos, Elza Tiemi Sakamoto-Hojo

**Affiliations:** 1 Department of Genetics, Ribeirão Preto Medical School, University of São Paulo—USP, Ribeirão Preto 14049-900, Brazil; 2 Department of Biotechnology, School of Arts, Sciences and Humanities—USP, São Paulo 03828-000, Brazil; 3 School of Pharmaceutical Sciences of Ribeirão Preto, University of São Paulo—USP, Ribeirão Preto 14040-900, Brazil; 4 Laboratory of Genetics and Molecular Biology, Department of Basic and Oral Biology, School of Dentistry of Ribeirão Preto, University of São Paulo, Ribeirão Preto 14049-900, Brazil; 5 Department of Biology, Faculty of Philosophy, Sciences and Letters at Ribeirão Preto, University of São Paulo—USP, Ribeirão Preto 14040-901, Brazil

**Keywords:** neurodifferentiation, neurite outgrowth, molecular docking, multi-target, cholinesterase activity

## Abstract

Alzheimer’s disease (AD) is a progressive and complex neurodegenerative disease. Acetylcholinesterase inhibitors (AChEIs) are a major class of drugs used in AD therapy. ROCK2, another promising target for AD, has been associated with the induction of neurogenesis via PTEN/AKT. This study aimed to characterize the therapeutic potential of a novel donepezil–tacrine hybrid compound (TA8Amino) to inhibit AChE and ROCK2 protein, leading to the induction of neurogenesis in SH-SY5Y cells. Experiments were carried out with undifferentiated and neuron-differentiated SH-SY5Y cells submitted to treatments with AChEIs (TA8Amino, donepezil, and tacrine) for 24 h or 7 days. TA8Amino was capable of inhibiting AChE at non-cytotoxic concentrations after 24 h. Following neuronal differentiation for 7 days, TA8Amino and donepezil increased the percentage of neurodifferentiated cells and the length of neurites, as confirmed by β-III-tubulin and MAP2 protein expression. TA8Amino was found to participate in the activation of PTEN/AKT signaling. In silico analysis showed that TA8Amino can stably bind to the active site of ROCK2, and in vitro experiments in SH-SY5Y cells demonstrate that TA8Amino significantly reduced the expression of ROCK2 protein, contrasting with donepezil and tacrine. Therefore, these results provide important information on the mechanism underlying the action of TA8Amino with regard to multi-target activities.

## 1. Introduction

Alzheimer’s disease (AD) is a progressive and complex neurodegenerative disease [1], conceptualized as a continuum from the preclinical, with Mild Cognitive Impairment, to the mild, moderate, and severe clinical stages of dementia [1,2]. AD accounts for about 60 to 70% of all dementia cases worldwide [3]. To date, only symptomatic treatments have been approved for AD, including acetylcholinesterase inhibitors (AChEIs) such as tacrine, donepezil, galantamine, and rivastigmine, in addition to an N-methyl-D-aspartate receptor antagonist, memantine [4]. Tacrine was the first drug approved by the Food and Drug Administration, but it was discontinued due to several side effects, including hepatotoxicity [5]. Other side effects induced by cholinergic drugs include nausea, vomiting, diarrhea, and tremors [6]. In this context, the search for new therapeutic modalities, which may be more effective for AD patients, is necessary and constitutes a major challenge for researchers, once the pathophysiology of AD is still poorly clarified.

The progressive memory impairment in AD patients has an impact on the daily life of affected individuals, and this condition has been closely associated with hippocampal degeneration [7]. There is evidence that new neurons can be generated throughout adult life in a process known as adult neurogenesis [8]; this is supported by the fact that neural progenitor cell (NPCs) niches exist in the dentate gyrus (DG) of the hippocampus and the subventricular zone (SVZ) of the lateral ventricles, and these cells can continuously generate new neurons throughout one’s lifetime [9,10]. It is important to note that newborn neurons generated in the SVZ of the adult brain can migrate for long distances through the rostral migratory stream (RMS) to the olfactory bulb (OB), where they become synaptically integrated into the existing circuitry, which is implicated in learning and memory processes related to the sensation of smell [10,11,12]. However, alterations in the neurogenesis mechanism may occur in the adult hippocampus, representing a critical early event in the course of AD development [13]. Interestingly, it has been demonstrated that the induction of adult hippocampal neurogenesis can improve cognitive function in AD patients [13,14], but the processes related to adult neurogenesis involve several crucial and complex steps. Furthermore, there are a variety of key molecules involved in the pathogenesis of AD that can impact the generation of new neurons [13].

The phosphatidylinositol 3-kinase (PI3K)/protein kinase B (AKT) signaling pathway plays an essential role in the development of brain structure, promoting the extension of neurites (dendrites and axons), in addition to acting in the regulation of neuronal synaptic plasticity, especially in the hippocampus. The activation of this pathway inhibits the autophagy process, promoting protection in hippocampal neurons of rat embryos [15]. Furthermore, the PI3K/AKT activation was correlated with increased cell survival, in part by phosphorylation and inhibition of death-inducing proteins, including Glycogen synthase kinase 3 (GSK-3), Bcl-xL/Bcl-2-associated death promoter (BAD), and caspase-9. Additionally, dysregulation of this pathway is commonly reported in the brains of AD patients [16].

An important negative regulator of the PI3K/AKT signaling pathway is the phosphatase and tensin homolog (PTEN) protein, which catalyzes the dephosphorylation of PI(3,4,5)P3 to PI(4,5)P2 (or PIP3 to PIP2), leading to the inhibition of this pathway [17]. The PI3K signaling pathway associated with PTEN function plays a relevant role in neurons during the control of cell growth, division, survival, and differentiation, to produce highly polarized neuronal morphologies with exquisite specializations, such as growth cones and synapses [18]. In addition, PTEN has been described as a tumor suppressor that inhibits cell growth and survival [19]. 

PTEN was identified as a substrate of the Rho-associated coiled coil containing kinases protein (ROCK). ROCK phosphorylates PTEN, which stimulates its phosphatase activity and thus negatively regulates the PI3K/AKT signaling pathway [20]. Two mammalian ROCK homologs, ROCK1 and ROCK2, have been identified. While ROCK1 is mainly expressed in non-neuronal tissue, ROCK2 is abundantly expressed in the brain and spinal cord, and its expression increases with age [21]. It has been demonstrated that ROCK is responsible for the caspase signaling cascades activation and cell death by apoptosis [20], and it also plays a role in the retraction and loss of neural processes and synapses, which mediates the flow and storage of information in the brain [19]. In studies carried out by Wu et al. [22], Fasudil, a potent inhibitor of ROCK protein, protected the brain against ischemic injury by decreasing neuronal apoptosis, thus revealing the association of ROCK inhibition with the activation of the PTEN/AKT pathway. Furthermore, another ROCK inhibitor, Y-27632, was found to modulate neurite outgrowth, protecting neurons against excitotoxicity-induced cell death [23]. In this sense, regarding therapeutic purposes, there is a growing interest in the search for new information at the molecular level of AD, which is relevant to investigate the role of ROCK2 and PTEN in cell function, particularly in neurons, once PI3K/AKT pathway is implicated in cell survival and also in negative regulation of cell death pathways [15]. 

In this study, we tested the hypothesis that the new AChEIs compound, TA8Amino, could be acting in the inhibition of acetylcholinesterase (AChE) and the ROCK2 protein, resulting in PTEN inactivation and, consequently, in activation of PI3K/AKT signaling, promoting neurodifferentiation and neuritogenesis in SH-SY5Y cells.

The present study provides important information on the characterization of the biological properties of TA8Amino, a new donepezil–tacrine hybrid compound, which is a multi-target dual inhibitor of AChE and ROCK2 protein. TA8Amino not only promotes an increase in cholinergic activity but also inhibits ROCK2, thus inducing the PI3K/AKT pathway, leading to neurogenesis and neuritogenesis of SH-SY5Y cells, in contrast to donepezil or tacrine. Therefore, these results support the mechanism underlying the action of TA8Amino related to multi-targeted activities, which is considered a potential therapeutic candidate for AD treatment.

## 2. Results

### 2.1. Neuronal Differentiation of SH-SY5Y Cells 

**Figure 1:**
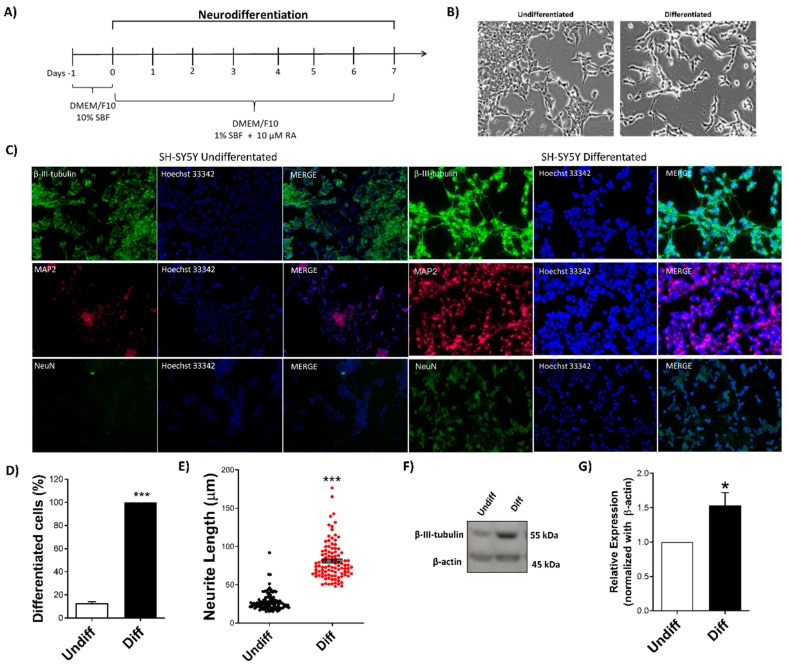
Characterization of SH-SY5Y neurodifferentiation by RA. (**A**) Protocol of neuronal differentiation: On day –1, cells were plated and cultured in a culture medium containing 10% FBS. After 24 h (day 0), the culture medium was removed and replaced by a new medium containing 1% FBS and 10 μM RA. The treatment medium was changed every 2 days and, after 7 days of treatment, cells were collected and analyzed. (**B**) Morphological changes were observed after treatment in the differentiated compared to the undifferentiated cells (20× magnification). (**C**) Immunofluorescence plates of SH-SY5Y cells undifferentiated and differentiated into neurons, showing positive labeling for anti-β-III-tubulin (1:500), anti-MAP2 (1:500), anti-NeuN (1:500), and Hoechst-33342 staining (0.15 μg/mL), visualized at 20× magnification in a Zeiss fluorescence microscope (Axio Imager). (**D**) Percentages of neurodifferentiated cells and (**E**) neurite length were analyzed by the ImageJ software (Figi). (**F**,**G**) Expression of β-III-tubulin (~55 kDa) and β-actin (~45 kDa) proteins analyzed by Western blotting before and after neuronal differentiation. Values were calculated by normalization with endogenous β-actin protein expression using the Image Studio Lite Ver 5.0 software (Lite Software). For all tests, at least three independent experiments were performed. Data were analyzed using Student’s *t*-test, being expressed as mean ± SEM. * *p* < 0.05; *** *p* < 0.001 indicate statistically significant differences compared to the control. SH-SY5Y Undifferentiated (HAM-F10/ DMEM (1:1) medium containing 10% FBS); SH-SY5Y differentiated (RA 10 µM).

SH-SY5Y cells were differentiated into neurons for 7 days after RA treatment (Figure 1A). At the end of neuronal differentiation, it was possible to observe morphological changes, such as reduction in the cytoplasm size, neurites growth, and cell proliferation reduction (Figure 1B). Once the morphological differentiation was confirmed, the next step was the detection of neuro-specific proteins to demonstrate the neuronal differentiation. The immunofluorescence assay was performed to detect NeuN protein, a universal neuron marker, which is exclusively expressed in nervous tissue, as well as the neurospecific proteins β-III-tubulin and MAP2. Undifferentiated SH-SY5Y cells were observed to weakly express neuronal differentiation markers. After differentiation with RA, NeuN-positive cells were detected in the perinuclear cytoplasm, and MAP2 and β-III-tubulin-positive cells were detected in the cytoplasm and neurites, respectively. Thus, AR induced differentiation of SH-SY5Y cells within the period of 7 days (Figure 1C).

The quantification of the percentage of differentiated cells into mature neurons and the evaluation of neuritogenesis by the quantification of neurites’ average size were performed. RA treatment was effective to differentiate 100% of cells (*p* < 0.001) when compared to the negative control (about 13% of spontaneously differentiated cells) (Figure 1D); a significant increase (*p* < 0.001) was observed in the neurite size (68.58 µm) measured in neurites formed after RA neurodifferentiation, compared to the negative control (18.29 µm) (Figure 1E). Neuronal differentiation was confirmed by quantification of β-III-tubulin protein expression. Differentiated cells showed a significant (*p* < 0.05) increase in β-III-tubulin expression compared to undifferentiated SH-SY5Y cells (Figure 1F,G).

### 2.2. TA8Amino Did Not Show Cytotoxic Effects in Undifferentiated and Differentiated SH-SY5Y Cells

**Figure 2:**
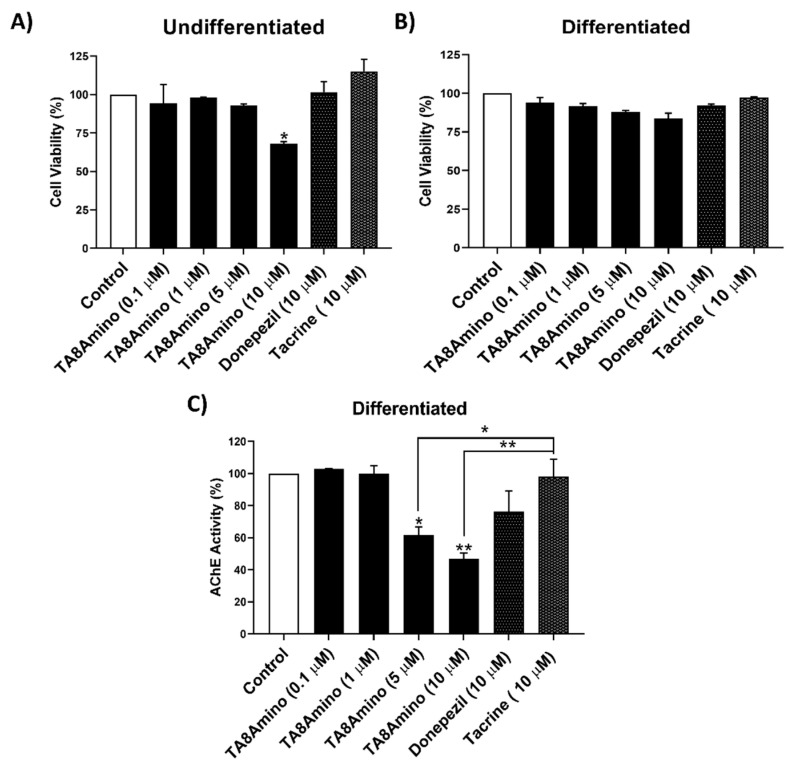
Cytotoxicity and AChE activity after treatment with AChEIs in undifferentiated and differentiated SH-SY5Y cells. Percentages of cell viability obtained for treatments with the TA8Amino compound in undifferentiated (**A**) and differentiated SH-SY5Y cells (**B**) after 24 h of treatment; cell viability was determined by XTT assay. AChE activity (%) was evaluated at different concentrations of TA8Amino compound, as well as 10 µM donepezil and 10 µM tacrine, in differentiated SH-SY5Y cells after 24 h of treatment (**C**). For all tests, at least three independent experiments were performed. Data were analyzed by One-Way ANOVA with Tukey’s post-test. Data are expressed as mean ± SEM. * *p* < 0.05; ** *p* < 0.01 indicate statistically significant differences compared to negative control and between groups. Control (HAM-F10/DMEM (1:1) medium containing 1% FBS).

A viability assay was performed for TA8Amino at different concentrations (0.1, 1, 5, and 10 µM) after 24h of treatment. Donepezil and tacrine were used as comparative control drugs at a concentration of 10 µM, as this concentration is the most commonly used in the literature [24]. In undifferentiated SH-SY5Y cells, there was a significant (*p* < 0.05) reduction in cell viability only for cells treated with 10 µM TA8Amino (67.8% viable cells), but for lower concentrations, TA8Amino did not show cytotoxic effects. Similarly, for Donepezil and Tacrine, significant differences were not observed in cell viability (Figure 2A). The results obtained with differentiated SH-SY5Y cells did not show significant differences in cell viability analyzed for all treatments, indicating that neuron-differentiated SH-SY5Y cells were more resistant to drug treatments compared to undifferentiated cells (Figure 2B). 

### 2.3. TA8Amino Inhibits AChE Activity at Lower Concentrations Than Donepezil or Tacrine

The analysis of AChE enzyme activity in differentiated SH-SY5Y cells was performed at different concentrations of TA8Amino (0.1; 1; 5 and 10 µM) after 24 h of treatment. The drugs donepezil and tacrine were tested at a concentration of 10 µM. At higher concentrations, they present cytotoxicity [24]. TA8Amino compound was able to significantly inhibit the activity of the AChE enzyme in a dose–response manner. Thus, an AChE activity of 61.73% (*p* < 0.05) and 46.9% (*p* < 0.01) was observed at concentrations of 5 and 10 µM, respectively, when compared to a negative control. Donepezil and tacrine (10 µM) did not induce significant changes in the percentage of AChE activity (Figure 2C). Such results demonstrated greater AChE inhibitory activity induced by the TA8Amino compound compared to the drugs donepezil or tacrine. Based on these results, the concentration of 5 µM was chosen to proceed with the assays.

### 2.4. TA8Amino Induces Neurodifferentiation and Neuritogenesis

**Figure 3:**
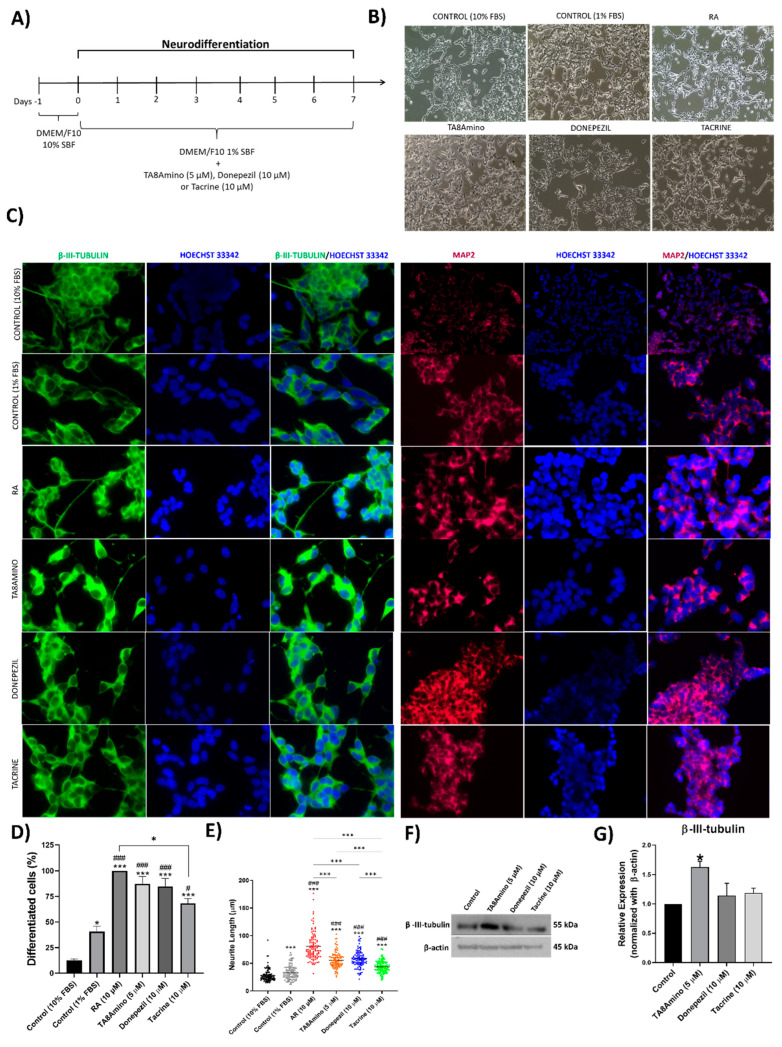
AChEIs induces neurodifferentiation in SH-SY5Y cells. (**A**) Protocol of neuronal differentiation: on day −1, cells were plated and cultured in culture medium containing 10% FBS. After 24 h (day 0), the culture medium was removed and replaced by a fresh medium containing 1% FBS with or without AChEIs. The treatment medium was replaced every 2 days and, after 7 days of treatment, cells were collected and analyzed. (**B**) Morphological analysis of SH-SY5Y cells after 7 days of neuronal differentiation with TA8Amino (20× magnification). (**C**) Immunofluorescence of SH-SY5Y cells treated with AChEIs for 7 days, showing anti-β-III-tubulin (1:500), anti-MAP2 (1:500) and nuclear Hoechst-33342 staining (0.15 μg/mL) visualized in a Zeiss fluorescence microscope (Axio Imager) at 40× magnification. (**D**) Percentages of neurodifferentiated cells and (**E**) Neurite lengths were assessed by the ImageJ software (Figi) (**F**,**G**). Expression of β-III-tubulin (~55 kDa) and β-actin (~45 kDa) proteins was analyzed by Western blotting before and after neuronal differentiation. Values of expression levels were calculated by normalization with the expression of β-actin protein (endogenous control) using the Image Studio Lite Ver 5.0 software (Lite Software). For all tests, at least three independent experiments were performed. Data were analyzed by the One-Way ANOVA test with Tukey’s post-test. Data are expressed as mean ± SEM. * *p* < 0.05; *** *p* < 0.001; indicates statistically significant differences compared to the control (10% FBS) and between groups. # *p* < 0.05; ### *p* < 0.001; indicates statistically significant differences compared to the RA.

SH-SY5Y cells were treated with AChEIs for seven days (Figure 3A). At the end of treatment, cells treated with TA8Amino and donepezil showed morphological changes, such as reduction in cytoplasm, neurite growth, formation of neuronal interconnection points, and reduction in cell proliferation, similar to the results seen for the RA treatment (positive control) (Figure 3B). These results demonstrate that the cells showed morphological characteristics typical of mature neurons after differentiation, which was confirmed by the expression of the neurodifferentiation markers β-III-tubulin and MAP2, located in the cytoplasm and neurites of the cells (Figure 3C).

Quantification of the percentage of neuronal differentiation was performed for the AChEI treatments, the controls with 10% FBS and 1% FBS, and treatment with RA (10 µM). It was observed that the decrease to 1% FBS in the culture medium was able to induce a significant increase (*p* < 0.05) in the percentage of neuronal differentiation (40.6%) when compared to the control with 10% FBS (12.6%). RA was able to induce 100% of differentiated cells when compared to controls (*p* < 0.001). Meanwhile, TA8Amino-treated cells differentiated into neurons showed 87% neurodifferentiation (*p* < 0.001), while donepezil induced 84% of neurodifferentiated cells (*p* < 0.001) compared to the 10% FBS control, as well as 1% FBS. Tacrine, on the other hand, showed an average of neurodifferentiated cells of 68% (*p* > 0.001) (Figure 3D). The average neurite size observed for the control (10% FBS) was 25.1 µm, whereas the control with 1% FBS showed a significant increase (*p* < 0.001) in neurite growth 34.45 µm. When AR was evaluated, an average neurite size of 67.3 µm was observed (*p* < 0.001). TA8Amino-treated cells demonstrated a significant increase (*p* < 0.001) in neurite size 55.81 µm. Similar results were observed for donepezil and tacrine, which showed a median neurite size of 57.62 µm (*p* < 0.001) and 43.41 µm (*p* < 0.001), respectively (Figure 3E). Neuronal differentiation was confirmed by the quantification of β-III-tubulin protein expression. Cells treated with TA8Amino showed a significant (*p* < 0.05) increase in β-III-tubulin expression compared to undifferentiated SH-SY5Y cells (control). On the other hand, donepezil and tacrine did not alter the β-III-tubulin protein expression profile (Figure 3F,G).

### 2.5. AChEIs Does Not Induce Changes in Cell Cycle Kinetics and Cell Death

**Figure 4:**
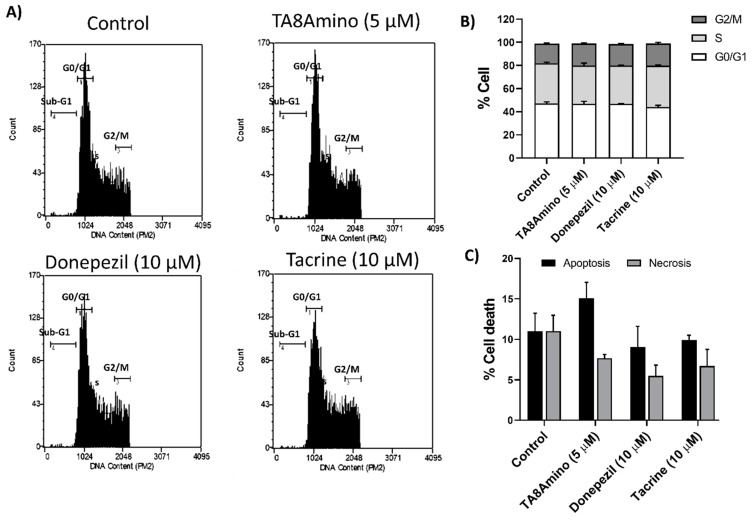
Cell cycle analysis after 7 days of treatment with AChEIs in SH-SY5Y cells. (**A**) Cell cycle analysis was performed by detecting and sorting cells with different embedded IP profiles (flow cytometry). (**B**) Distribution of cells at different phases of the cell cycle after treatment with AChEIs in SH-SY5Y cells. (**C**) Percentage of apoptotic and necrotic cells obtained by flow cytometry using the eBioscience™ Annexin V Apoptosis Detection Kits (Invitrogen) reagent. Values were obtained from at least three independent experiments performed in triplicate and expressed as mean ± SEM. Control (HAM-F10/DMEM (1:1) medium containing 1% FBS).

It was observed that all treatments performed with the AChEIs compounds did not cause significant changes in the distribution of cells in the cell cycle phases under the treatment conditions (Figure 4A,B). As well, none of the treatments performed with AChEIs compounds induced significant changes in the percentage of apoptosis and/or necrosis after 7 days of treatment (Figure 4C). 

### 2.6. TA8Amino Modulates Akt Gene Expression

**Figure 5:**
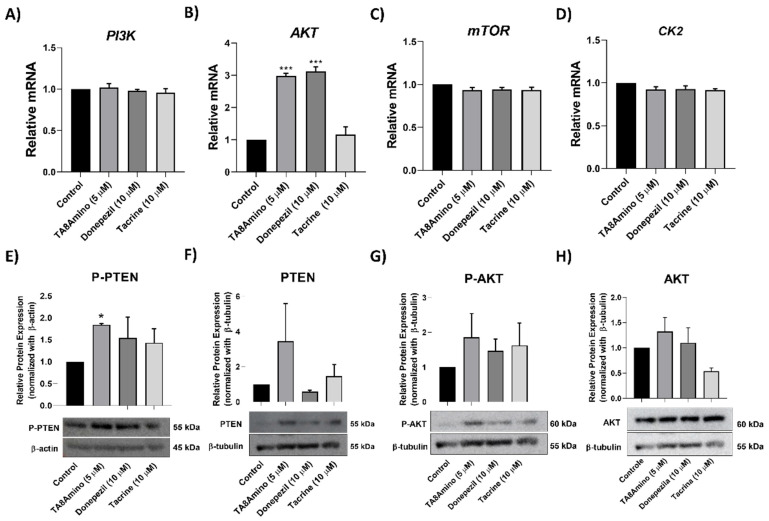
Gene and Protein expression levels in SH-SY5Y cells treated with AChEIs compounds for 7 days. Relative expression of mRNA *PI3K* (**A**), *AKT* (**B**), *mTOR* (**C**), and *CK2* (**D**) was analyzed by qPCR. (**E**) Phospho-PTEN (Ser380/Thr382/383) protein expression (∼55 kDa); (**F**) total PTEN (∼55 kDa); (**G**) Phospho-AKT (Ser473) (∼60 kDa); (**H**) total AKT (∼60 kDa). The values were normalized with the endogenous β-actin protein (∼45 kDa), using the Image Studio Lite Ver 5.0 program (Lite Software). Data were analyzed using One-Way ANOVA test with Tukey’s post-test. Values were obtained from at least three independent experiments and expressed as mean ± SEM. * *p* < 0.05; *** *p* < 0.001 indicates statistically significant differences when compared to the control. Control (HAM-F10/DMEM (1:1) medium containing 1% FBS).

Once we demonstrated that TA8Amino and donepezil act by inducing neurogenesis, we investigated the molecular mechanisms that regulate adult neurogenesis, based on the information that the endothelial Akt pathway can contribute to adult hippocampal neurogenesis and cognitive functions [25,26]. We evaluated the transcript expression levels of *PI3K*, *AKT*, *mTOR*, and *CK2* genes after AChEIs treatment for 7 days (Figure 5A–D). We observed a significant upregulation (3-fold; *p* < 0.001) of *AKT* gene for cells treated with TA8Amino and donepezil (but not tacrine) compared to the control (Figure 5B). Changes in expression levels were not observed for *PI3K*, *mTOR*, or *CK2*. 

### 2.7. TA8Amino Induces the PTEN/AKT Pathway

The expression of proteins involved in the PTEN/AKT signaling pathway was evaluated by Western Blotting after 7 days of neuronal differentiation with AChEIs in SH-SY5Y cells. It was possible to observe a significant increase (*p* < 0.05) in the phospho-PTEN(Ser380/Thr382/383) in cells treated with the hybrid compound, TA8amino, compared to the control (Figure 5E). It was also observed that TA8Amino induced a non-significant increase in total PTEN expression (*p* < 0.1) (Figure 5F). Thereafter, a non-significant increase in phosphorylated and total AKT induced by TA8Amino was observed (Figure 5G,H).

### 2.8. Preparation of the ROCK2 Framework

The absent residues in the structure of the ROCK2 protein (6ED6—Chain A), 266–269, and 388–394 were modeled using the loop model protocol of the Modeller software [27]. The ROCK2 structure was submitted to MD simulation in triplicate of 100 ns to verify whether it would present satisfactory structural stability to be used in the docking study with the TA8amino compound (Figure S1). The system is a cubic type, composed of protein, water molecules (TIP3), and ions, characterizing the cellular environment.

The analysis of the graph generated for the RMSD of the ROCK2 structure showed a similar trajectory throughout the three 100 ns MD simulations, with a maximum variation of approximately 4 Å throughout the simulations. The analysis of the graph generated for the RMSF of the structure showed that, in the N-terminal region, there was an increase (approximately 10 Å) in the RMSF for the first ten amino acids concerning the others, showing an increase of approximately 2 Å of amino acids 380 to 390 when compared to others. The analysis of the structure compaction (contraction and expansion) over time presented in the graph of the turning radius (Rg) showed that, to its initial compaction state, the structure of ROCK2 presented variations of approximately 1.5 Å after the first 10 ns of simulation and this oscillation in its compacted state remained until the end of the simulation at 100 ns (Figure S2).

As a resource to analyze the regions with the greatest atomic displacement of the protein, an analysis was performed using the b-factor, which confirmed that the location of the regions with the greatest structural variation by amino acids observed in the RMSF graph corresponds, predominantly, to the N-terminus and the loops (Figure S3). The greatest structural variations observed in RMSF are often associated with loops, as they are structures with the main function of connecting secondary elements and providing the structural flexibility necessary for the protein to perform its function [28,29,30,31]. The analysis of the binding site region, where the amino acids K121, E127, F137, and M172 are located, showed low structural flexibility as observed in the b-factor analysis (Figure S4), indicating that this is a suitable place to use as a therapeutic target.

### 2.9. TA8amino Docking with ROCK2

The docking of the TA8amino compound [32] was directed to the ROCK2 binding site located in the protein kinase domain, which comprises amino acids 92 to 354, specifically in the region of amino acids K121, E127, F137, and M172 [33]. The best pose among the 10 conformations obtained was chosen according to the energy score of the bonds and was used as input for MD simulation studies.

**Figure 6:**
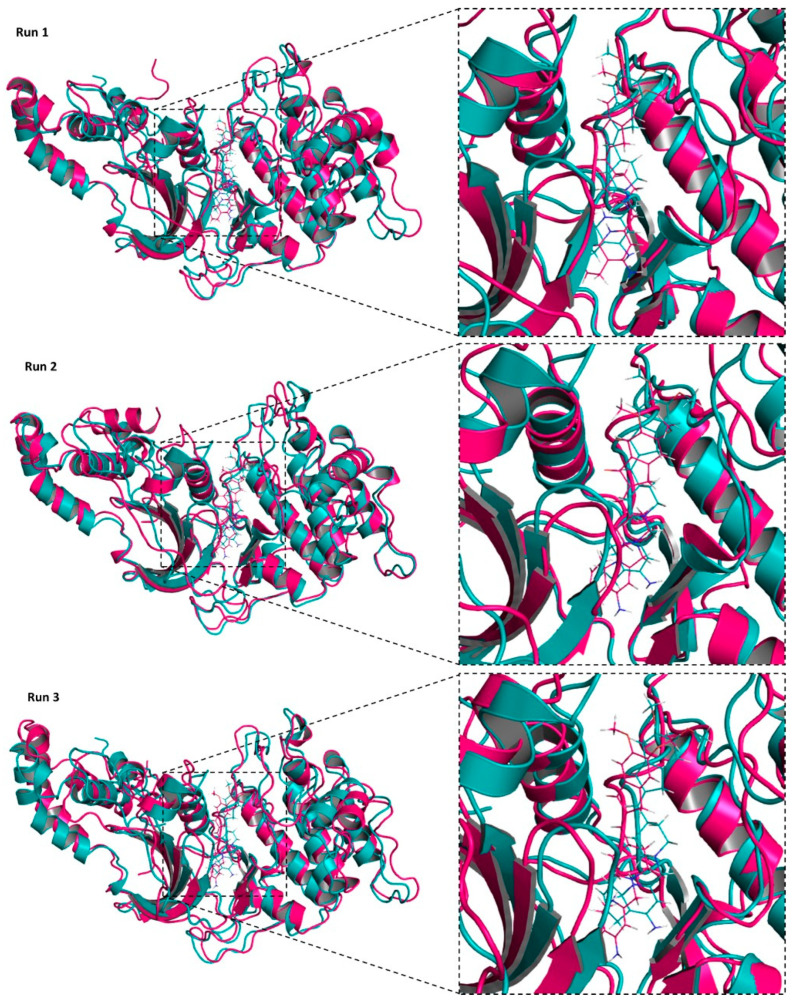
Alignment of ROCK2/TA8amino complexes in the initial and final times of MD simulation.

The ROCK2/TA8amino (R2/TA8) complex obtained by molecular docking was submitted to MD simulations in triplicate, with a duration of 100 ns per each simulation. The trajectories of the simulations were analyzed based on RMSD, RMSF, b-factor, and alignment of structures over the time of MD simulation. Alignment of the initial (0 ns) and final (100 ns) structures showed that the TA8amino compound remained at the ROCK2 binding site throughout the three MD simulations lasting 100 ns. This was observed in the triplicates performed for the R2/TA8 complex (Figure 6), indicating that it may be interesting to evaluate the action of the TA8amino compound on ROCK2 under experimental in vitro conditions.

Alignment of the initial R2/TA8 complexes at 0 ns (blue) and final at 100 ns (pink) of the three MD simulations. The black dotted rectangles highlight the regions of the ROCK2 binding site where the TA8amino compound was docked. The images show that TA8amino remained at its binding site throughout the MD simulation time in the three simulations performed.

**Figure 7:**
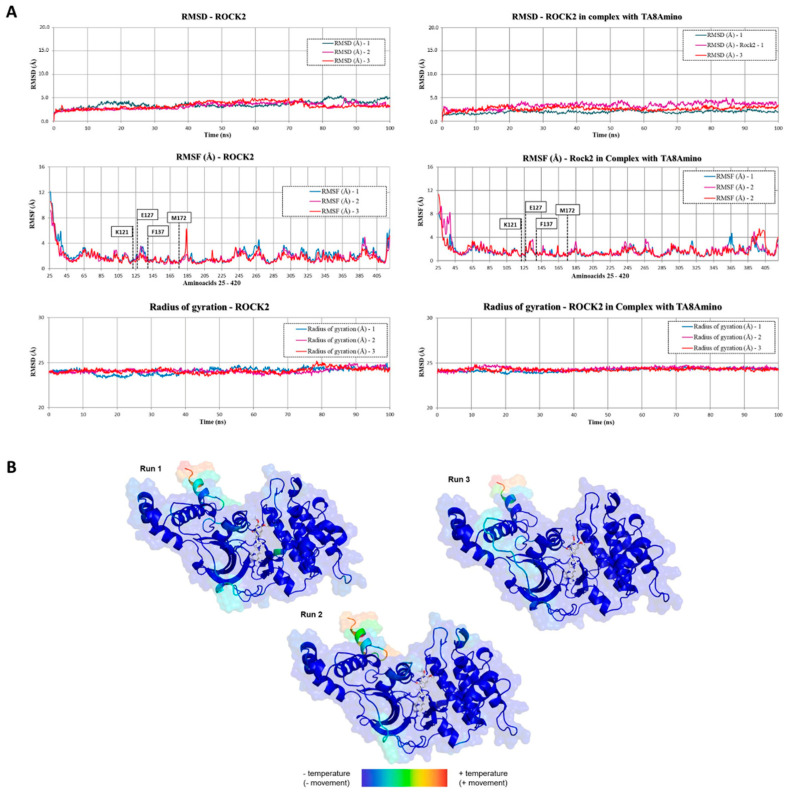
Comparison of structures throughout the MD simulation. (**A**) Values of RMSD, RMSF, and radius of rotation of the coordinates of the Cα atoms as a function of the time interval of the triplicate simulations of ROCK2 alone and ROCK2 in complex with TA8Amino. (**B**) Results of the MD simulation b−factors of the R2/TA8 complex performed in triplicate. The region of the binding site where the TA8amino compound is anchored shows that there was little variation in temperature and movement.

The analysis of the graphs generated for the RMSD of the R2/TA8 complex showed that the ROCK2 protein in complex with the compound TA8amino presented structural behavior with less variation when compared to the triplicate of simulation of MD without the compound TA8amino docked in its site. In a complex with TA8amino, ROCK2 presented trajectories that varied between 1.8 Å and 4.5 Å over the three simulations, while ROCK2 without TA8amino presented variations between 2.2 Å and 5.2 Å over three simulations. Thus, it can be concluded that TA8amino promoted less structural movement toward ROCK2 (Figure 7A).

The analysis of the structure compaction (contraction and expansion) over time presented in the graph of the turning radius (Rg) showed that, concerning its initial compaction state, the structure of ROCK2 in complex with TA8amino presented variations, approximately 0.8 Å, after the first 25 ns of simulation, and this oscillation in its compacted state remained until the end of the simulation at 100 ns. When compared with the Rg results obtained for the structure of ROCK2 without TA8amino docked in its site, it was observed that the ROCK2 structure in complex with TA8amino showed less oscillation in its contraction and expansion states throughout the MD simulation (Figure 7A).

The analysis of the graph generated for the RMSF of the ROCK2 structure in complex with the compound TA8amino showed that, for the three MD simulations, the structural behavior was similar to that observed for ROCK2 without TA8amino. This result was also observed in the b-factor analysis. The region corresponds to the binding site where TA8amino was docked and interacted with ROCK2 throughout the MD simulation and showed high stability in both RMSF and b-factor analysis (Figure 7B). 

Molecular MD simulations performed with the R2/TA8 complex showed that the TA8amino compound was stable at the target site throughout the 100 ns MD simulation triplicate.

### 2.10. TA8Amino Induces Changes in ROCK2 Protein Expression

**Figure 8:**
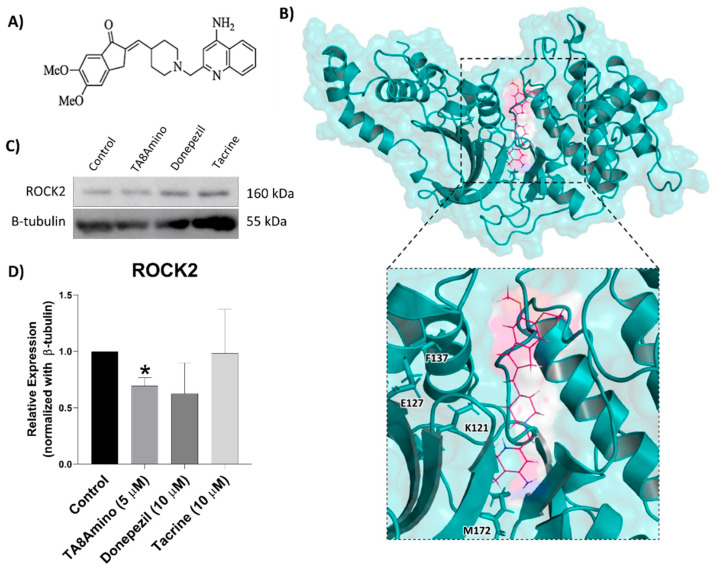
Effect of TA8Amino on ROCK2 protein. (**A**) Chemical structure of the TA8Amino compound. (**B**) The protein kinase domain, which comprises amino acids 92 to 354 of the ROCK2 structure, is represented in blue, while TA8amino compound docked at the site is shown in pink. (**C**,**D**) Expression of ROCK2 (~160 kDa) and β-tubulin (~55 kDa) proteins were analyzed by Western blotting after 7 days of TA8Amino, donepezil, and tacrine treatments. Expression values were calculated by normalization to the endogenous β-tubulin protein expression using the Image Studio Lite Ver 5.0 software (Lite Software). Three independent experiments were performed, and the results were analyzed by the One-Way ANOVA test with Tukey’s post-test. The results are expressed as mean ± SEM. * *p* < 0.05 indicate a statistically significant difference compared to the control. Control (HAM-F10/ DMEM (1:1) medium containing 1% FBS).

Once we had demonstrated that TA8Amino is stable in the active site of ROCK2 throughout the MD simulation, we analyzed the effect of TA8Amino on ROCK2 protein expression in vitro after 7 days of treatment in SH-SY5Y cells. It was observed that TA8amino significantly (*p* < 0.05) reduced (about 30%) the expression of ROCK2 compared to the control. Donepezil and tacrine did not change the ROCK2 expression (Figure 8). Thus, it was possible to demonstrate that TA8Amino exerts influence on the ROCK2 protein expression.

## 3. Discussion

The main therapy for AD is based on the use of AChEIs [34]. AChEIs act by preventing the hydrolysis of acetylcholine (ACh) and activating the cholinergic system [35]. AChE has long been a viable therapeutic target to improve AD symptoms [34]. It is known that the AChEIs effects are limited to the symptoms of AD, and the drugs do not effectively stop or delay the progression of the disease [36]. Since AD has a multifactorial and complex nature, treatments that are based on a single molecular target do not show promising effects in slowing the progression of AD. Thus, the search for and discovery of molecules called multi-targeted ligands (MTDLs) has attracted scientists worldwide [19]. MTDLs, in addition to acting on different targets at the same time, have the advantage of avoiding the simultaneous administration of several drugs, facilitating their use by the patients [37].

In previous studies, it was demonstrated that the donepezil–tacrine hybrid compound (TA8Amino) showed a potent allosteric modulator of AChE and induced promising effects in terms of neuroprotection and neurodifferentiation [32,38]. These studies highlight that TA8Amino demonstrated low toxicity and a potent inhibitory effect on AChE at lower concentrations than donepezil and tacrine. It also induced neuritogenesis and neurodifferentiation in the SH-SY5Y lineage, as well as the ability to activate the PTEN/AKT pathway by inhibiting the ROCK2 protein, which is considered the mechanism associated with non-cholinergic effects.

In a previous report, the authors found that TA8Amino acts by allosterically inhibiting AChE, with an IC50 = 0.014 μM for hAChE of (hybrid 14), while donepezil is a mixed reversible competitive and non-competitive inhibitor (IC50 = 0.0057 μM), while tacrine is a reversible and non-competitive inhibitor (IC50 = 0.23 μM) [32,36]. Here, we performed the analysis of AChE inhibitory activity by treating SH-SY5Y cells with several concentrations of TA8Amino for 24h, compared to donepezil and tacrine at 10 μM (non-cytotoxic maximum value). We showed that TA8Amino induced biological inhibition of AChE with an IC50 of approximately 6 μM, whereas donepezil and tacrine did not significantly inhibit AChE at a similar concentration. In addition, it has been reported that donepezil and tacrine tested at concentrations above 10 μM were cytotoxic to neurons derived from SH-SY5Y cells [39,40]. Thus, we demonstrated that TA8Amino is an effective inhibitor of AChE, and it is less cytotoxic compared to donepezil and tacrine.

Studies have shown that the drug donepezil enhances neurite outgrowth in primal cortical cultures, whereas tacrine does not exert the same effects [41]. Furthermore, donepezil can increase hippocampal neurogenesis as well as induce greater neuronal survival in an animal model [42]. Thus, these data corroborate our findings, since we demonstrated that TA8Amino also induces neurogenesis and neuritogenesis in SH-SY5Y cells, indicating similar results to those observed for donepezil. It is known that in AD, the accumulation of hyperphosphorylated tau protein causes a great impact on neurons, leading to a reduction in the number of synapses, a decrease in adult neurogenesis, and consequent neurodegeneration [43]. In this sense, the investigation of adult neurogenesis has become an interesting and attractive approach to the development of therapies for patients with neurodegenerative diseases [13]. Several studies have shown that the induction of adult hippocampal neurogenesis by various agents, such as erythropoietin [44,45], fluoxetine [46], brain-derived neurotrophic factor [47], curcumin [48], and selenomethionine [49], among others, have become promising treatment strategies for AD [49,50,51,52].

In this study, we also analyzed the capacity of TA8Amino to influence the PI3K/AKT signaling pathway. A significant increase in *AKT* gene and protein expression was observed in the experiments performed with TA8Amino and donepezil in SH-SY5Y cells. Several studies demonstrate that donepezil exerts neuroprotective effects from the activation of α4 and α7 nAChRs and the PI3K-AKT pathway [53,54]. There is evidence that PTEN, which is a negative regulator of this pathway, prevents axon regeneration [55], and the pharmacological inhibition of PTEN promotes axonal elongation, neuronal differentiation, and increases axon regeneration in both the CNS and the PNS [56,57]. PTEN can be activated by multiple kinases, including CK2, GSK3β, and ROCK2 kinase, that phosphorylate several residues (S229, T232, T319, and T321) in the C2 domain [57,58]. ROCK2 acts on actin cytoskeleton dynamics, and this position can be an interesting drug target [59]. 

The inhibition of ROCK2 is known to promote neurogenesis and neuritogenesis in several neuronal models [60]. Therefore, we performed in silico analysis to investigate whether TA8Amino would be able to inhibit ROCK2. The MD simulations performed with the R2/TA8 complex showed that the TA8amino compound was stable at the site used as a target, demonstrating that TA8amino has an affinity for interaction with ROCK2. The analysis of ROCK2 protein expression in SH-SY5Y cells treated with TA8amino showed a significant reduction in its expression, but the same result was not observed for cells treated with donepezil or tacrine. In studies with Fasudil, a ROCK inhibitor, the authors observed an in vitro induction of neurogenesis in mouse neural stem cells [61], and also protection against oxygen–glucose deprivation in primary neurons of the hippocampus of mouse embryos [62]. Similar results were also observed for another ROCK inhibitor, Y-27632, which induced neurite outgrowth in PC12 cells by activating AKT phosphorylation cascades [63]. In another study, a benzofuran derivative (MBPTA), a ROCK inhibitor, protected SH-SY5Y cells against induced-oxidative stress, possibly through PI3K/Akt activation [64]. In our results, we showed that TA8Amino might inhibit ROCK2, preventing PTEN activation and, consequently, inducing the AKT signaling pathway, increasing neurogenesis and neuritogenesis in SH-SY5Y cells.

## 4. Materials and Methods

### 4.1. Chemicals

The compound TA8Amino (donepezil–tacrine hybrid) and the drugs donepezil and tacrine were provided by Prof. Ivone Carvalho, Medicinal Chemistry Laboratory, Faculty of Pharmaceutical Sciences at Ribeirão Preto—USP. TA8Amino was synthesized based on the hybridization of donepezil and tacrine chemical structure; it is a potent allosteric modulator of AChE capable of fixing a specific AChE conformation, confirmed by Saturation transfer difference (STD) Nuclear Magnetic Resonance (NMR) and molecular modeling studies [32]. All compounds were purified by flash chromatography with dichloromethane: methanol gradient (0–15%), showing a high degree of purity, as previously reported by Chierrito et al. [32]. The compounds AChEIs were dissolved in phosphate-buffered solution (PBS) at a concentration of 100 µM and stored at −20 °C

### 4.2. Cell lines and Treatment Conditions

SH-SY5Y cell line (human neuroblastoma) was purchased from Rio de Janeiro Cell Bank (CBRJ). Cells were grown in 25 cm^2^ cell culture flasks in culture medium composed of DMEM and HAM F10 (1:1) (Sigma-Aldrich, St. Louis, MO, USA) supplemented with 10% fetal bovine serum (FBS; Gibco, Grand Island, NY, USA), penicillin solution at 1% (100 units/mL) and 1% streptomycin (10 mg/mL) (Sigma-Aldrich). Cells were incubated at 37 °C in 5% CO_2_ until reaching the semi-confluency state (80%) when they were subcultured for experimental assays. The cell treatments varied according to each assay. In general, cells were treated for 24 h or 7 days. Each experiment was repeated at least three times. HAM F10 and DMEM medium (1:1) containing 1% FBS was used as a negative control.

### 4.3. Neuronal Differentiation 

Neuronal differentiation was performed in SH-SY5Y cells using RA (retinoic acid, 10 uM) following the protocol of Kunzler et al. [65]. Briefly, cells were treated with RA in HAM F10 and DMEM (1:1) medium containing 1% FBS for 7 days. The medium, with RA (10 µM) and 1% FBS, was changed every 2 days until completion of the differentiation period (7 days). For AChEIs-induced neuronal differentiation assays, the same protocol was performed (except for the addition of RA). The analysis of the percentage of neuronal differentiation and neurite outgrowth was performed according to a previous protocol [38]. Briefly, a total of 20 photographs (20× magnification) were taken of each group treatment using an inverted phase microscope (Zeiss, Jena, Germany) to quantify the percentage of differentiated cells and neurite size; the ImageJ software (Fiji, image processing package) and the Simple Neurite Tracer plugin were used for these measurements. Differentiated cells were considered for those that had at least one neurite larger than the cell diameter; a total of 100 cells per treatment and in experimental triplicate were analyzed. In addition, the neurite sizes of these cells were quantified, considering only one neurite per cell.

### 4.4. Immunofluorescence

The cells were fixed with methanol (Supelco-107018) for 10 min, then washed three times with PBS and permeabilized (PBS-Triton X-100 0.2%; Sigma-Aldrich) for 15 min. Cells were blocked with 2% PBS with bovine serum albumin (BSA; Sigma-Aldrich) solution for 1 h, following incubation in 2% PBS-BSA containing the primary antibodies: anti-β-III-tubulin rabbit (1:500 dilution; Abcam, Cambridge, Reino Unid, ab18207), anti-MAP2 rabbit (1:500 dilution; Abcam ab183830) and, anti-NeuN rabbit (1:500 dilution; Abcam ab104225) for 60 min at room temperature. Next, cells were washed three times with 0.1% PBS-Tween20 (Sigma-Aldrich) and incubated with Alexa Fluor^®^ 488 anti-rabbit secondary antibody or Alexa Fluor^®^ 651 anti-rabbit (Invitrogen, Life Technologies, Carlsbad, CA, USA) diluted 1:200 (1% PBS-BSA) for 30 min and washed again with 0.1% Tween20-PBS. Cells were stained with Hoechst 33342 (0.15µg/mL; Sigma-Aldrich) and analyzed under a fluorescence microscope (ZEISS Axio Imager, Jena, Germany).

### 4.5. Cytotoxicity Assay

The analysis of cytotoxicity was performed with Cell Proliferation Kit II—XTT (Roche Molecular Biochemicals, Mannheim, Germany). Cytotoxicity was evaluated in neuronal cells differentiated at different concentrations of the tested compounds. Cells were seeded in 24-well plates and treated with the compounds for 24 h. Then, cells were incubated with XTT solution according to the manufacturer’s instructions. The absorbance was measured at 492 and 690 nm (Epoch Microplate Spectrophotometer, BioTek, Winooski, VT, USA), and the final result for each sample is directly proportional to the number of viable cells. The value obtained for the negative control was considered 100%.

### 4.6. AChE Activity 

For the analysis of AChE activity, differentiated SH-SY5Y cells were treated with different concentrations of TA8Amino (0.1, 1, 5, and 10 µM), donepezil (10 µM), and tacrine (10 µM). After 24 h of treatment, the analysis was performed with Acetylcholinesterase Activity Assay Kit (Sigma-Aldrich) according to the manufacturer’s protocol.

### 4.7. Cell Death Analysis

The analysis of cell death induction was performed with eBioscience™ Annexin V Apoptosis Detection Kits (Invitrogen, Waltham, MA, USA) evaluated by flow cytometry, according to the manufacturer’s protocol, using Guava EasyCyte MiniSystem device (Guava Technologies, Hayward, CA, USA) and analyzed by Guava CytoSoft 4.2.1 Software Environment (Guava Technologies). Cells were analyzed after 7 days of treatment, and the results were presented as percentages of Annexin-V positive cells. In addition, necrotic cells were also counted in the same assay.

### 4.8. Analysis of Cell Cycle Kinetics

For cell cycle kinetics analysis, after 7 days of treatment, cells were fixed in iced 70% ethanol and kept at −20 °C until reading on the flow cytometer. For reading, samples were resuspended in 200 μL of propidium iodide solution (5 µg/mL; Sigma-Aldrich), incubated for 30 min at room temperature, and analyzed in Guava CytoSoft 4.2.1 Software Environment (Guava Technologies) flow cytometer, counting at least 5000 events for each sample. The values were expressed as percentages of cells distributed at each phase of the cell cycle.

### 4.9. RNA Extraction and qPCR

RNA was extracted from SH-SY5Y cells after the 7-day treatment with TA8amino, donepezil, and tacrine, using Trizol reagent (Invitrogen) according to the manufacturer’s instructions. RNA yield was assessed using a NanoDrop ND-1000 Spectrophotometer (Uniscience, São Paulo, Brazil), and RNA integrity was evaluated using Agilent RNA Nano 6000 chips onto Agilent 2100 Bioanalyzer (Agilent Technologies, Santa Clara, CA, USA). Only RNA samples with RNA integrity number (RIN) > 7 were considered for gene expression analysis. One microgram of RNA was reverse transcribed using the High-Capacity cDNA Reverse Transcription Kit (Thermo Fisher Scientific Inc., Waltham, MA, EUA) after DNase (Invitrogen) treatment following the manufacturer’s protocol. Real-time PCR was carried out using PowerUp™ SYBR™ Green Master Mix (Invitrogen), 30 ng of cDNA, and 750 nM of gene-specific primers (IDT Technologies, Coralville, Iowa, EUA): *PI3K* forward, 5′-GATACAGCAGACGGGACCTT-3′ and reverse, 5′-AGGTTAATGGGTCAGAGAAGC-3′; *AKT* forward, 5′-CCAAACTGTCCTCACCCTAT-3′ and reverse 5′-TGGAAGGAAGCCCTAGTAAG-3′; *mTOR* forward, 5′-GGACCACAGTGCCAGAATCT-3′ and reverse, 5′-CATGAGAGAAGTCCCGACCA-3′; *CK2* forward, 5′-TGAAGGACCAGGCTCGAATG-3′ and reverse, 5′-GCACTGAAGAAATCCCTGACAT-3′; *HPRT1* forward, 5′-TGGACAGGACTGAACGTCTT-3′ and reverse 5′-GAGCACACAGAGGGCTACAA-3′; *B2M* forward, 5′-AGGCTATCCAGCGTACTCCA-3′ and reverse, 5′-TCAATGTCGGATGGATGAAA-3′. qPCR reaction was carried out in 96-well plates sealed with MicroAmp^®^ Optical Adhesive Film in QuantStudio 3 equipment (Applied Biosystems, Waltham, MA, USA) using the following conditions: 50 °C for 2 min, 95 °C for 2 min, and 40 cycles of 95 °C for 1 s and 60 °C for 30 s. Primers were designed and verified against self-dimers or cross-dimers by analysis of melting curves and gel electrophoresis, showing one peak and single bands. The relative quantification of each target was obtained according to the 2^−∆∆Ct^ method [66] after normalization with HPRT1 and B2M genes.

### 4.10. Protein Expression

Proteins were extracted using a RIPA Lysis and Extraction Buffer solution (Thermo Fisher Scientific Inc.) with 1% HaltTM Protease and Phosphatase Inhibitor Cocktail (Thermo Fisher Scientific Inc.) with protease inhibitors; the quantification was performed in a spectrophotometer using BCA kit (Pierce, Waltham, MA, USA) and stored at −80 °C, until the next step for the analysis of protein expression by Western blot, according to the protocol described by Montaldi et al. (2015). The antibodies used were: anti-AKT rabbit (1:1000 dilution; Cell Signaling Technologies, Danvers, Massachusetts, EUA, #9272), anti-phosphor-AKT rabbit (Ser473) (1:1000 dilution; Cell Signaling Technologies #9271), anti-PTEN rabbit (1:1000 dilution; Cell Signaling Technologies #9556), anti- phospho-PTEN rabbit (Ser380/Thr382/383) (1:1000 dilution; Cell Signaling Technologies #9554), anti-ROCK2 rabbit (1:1000 dilution; Sigma Aldrich PLA0013), anti- β-III-tubulin rabbit (1:1000 dilution; Abcam ab18207), in addition to the endogenous anti- β-Actin rabbit (1:1000 dilution; Cell Signaling Technologies #4967), or anti-β-tubulin rabbit (1:1000 dilution; Cell Signaling Technologies #2146), used as a control for normalization. To visualize protein bands, the membranes were scanned using the ImageQuant LAS 500 (GE Healthcare Life Sciences, Chicago, IL, USA) and the intensity of bands was quantified by Image Studio Lite Ver 5.0 program (LI-COR Biosciences GmbH, Bad Homburg, Germany), the values being normalized concerning the endogenous protein.

### 4.11. In Silico Structural Analysis

#### 4.11.1. Preparation of the ROCK2 Framework

For the docking of TA8amino with ROCK2, the structure of ROCK2 determined by X-ray diffraction crystallography (PDB ID: 6ED6—Chain A) was used [33], which comprises amino acids 27 to 415, spanning the Protein kinase domain (amino acids 92–354). The missing residues in the structure of ROCK2 (6ED6—Chain A), 266–269, and 388–394 were modeled using the MODELLER 9.21 software, through the loop model protocol [27]. The final model, after refinement, was selected based on the Discrete Optimized Protein Energy (DOPE) score [67] and the QMEAN score [68].

#### 4.11.2. TA8amino Docking with ROCK2

TA8amino docking [32] at the ROCK2 binding site located in the region of amino acids K121 (lysine), E127 (glutamine), F137 (phenylalanine), and M172 (methionine) [33], was performed using AutoDock Vina software [69] and AutoDock Tolls package [70]. The same software was used for docking validation by redocking the co-crystallized ligand present in the 6ED6 structure of ROCK2 [33].

TA8amino in ROCK2 docking was preceded by the ligand and protein preparation step, where polar hydrogen atoms were added to the protein. The smallest possible search space was specified, which presented dimensions of 14 Å, 14 Å, and 14 Å, respectively, in the X, Y, and Z axes, around the ligand after redocking. This space encompassed active residues K121, E127, F137 and M172 present in ROCK2 binding site. The 10 poses obtained by docking were ranked by the energy score of the bonds in kcal/mol. The best pose was selected and used as input for molecular dynamics simulation studies.

#### 4.11.3. Molecular Dynamics (MD) Simulation of the Protein/Ligand Complex

MD simulations were performed using Gromacs 2019.3 software (GROMACS, Institute of Technology and Uppsala University, Sweden) [71]. Protein topology and structure files were prepared using CHARMM36 force field (Martin Karplus, Department of Chemistry, Harvard University) [72]. The ligand topology files were generated through the CGenFF server (Martin Karplus, Department of Chemistry, Harvard University) [73] using the CHARMM36 force field [72]. The water model used as solvent was TIP3P [74]. A cubic box was built around the protein–ligand complex, and the system was neutralized by adding an appropriate number of Na+ and Cl- ions, considering an ionic concentration of 0.15 M.

The systems were minimized and balanced under a canonical NVT set (number of particles, volume, and temperature) followed by an isothermal–isobaric NPT set (number of particles, pressure, and temperature). During the NVT and NPT equilibrium step, the protein and ligand positions were kept restricted. The production phase of the MD simulation was performed at 310 K for 100 ns in triplicate, without position restrictions. The root means square deviation (RMSD), root mean squared fluctuation (RMSF), the radius of gyration (Rg), and b-factor tools were used for the analysis of the trajectories.

### 4.12. Statistical Analysis

For the different assays, the results were presented as mean values ± Standard Error (SEM) from at least three independent experiments. For analyses with independent samples, a *t*-test was always used in comparison to the control. For more than three experimental groups, we used One-Way ANOVA with Tukey’s post-test. GraphPad Prism v.9 (GraphPad Software, Inc. San Diego, CA, USA) was used to perform statistical analysis and construction of graphs, and *p* < 0.05 was considered the limit for a statistically significant difference between groups.

## 5. Conclusions

In conclusion, these results provide important information about the characterization of the biological properties of TA8Amino with regard to its molecular interactions (multi-target activities), since the compound not only promotes an increase in cholinergic activity (through the inhibition of AChE), but also inhibits ROCK2, thus modulating the PTEN/AKT pathway, leading to neurogenesis, which is an interesting effect. As a whole, the donepezil–tacrine hybrid compound (TA8Amino) may be considered a potential therapeutic candidate to be investigated for the treatment of AD.

## Data Availability

The data contained in this study are available from the authors upon reasonable request. The raw data that support the conclusions of this manuscript will be made available by the authors, without undue reservations, to any qualified researcher.

## Supplementary Materials

The following supporting information can be downloaded at: https://www.mdpi.com/article/10.3390/ijms232314788/s1, Figure S1: Structure of the complete ROCK2 protein; Figure S2: Trajectory of the DM simulation of the structure of ROCK2; Figure S3: Alignment of ROCK2 structures at the start and end time of the DM simulation; Figure S4: Structural behavior observed by the b-factor.
