# Supplementary material for: Novel Dual AChE and ROCK2 Inhibitor Induces Neurogenesis via PTEN/AKT Pathway in Alzheimer’s Disease Model
Source: Int J Mol Sci. 2022 Nov 26;23(23):14788. doi: 10.3390/ijms232314788 (PMC9737254; doi:10.3390/ijms232314788)
Supplement: Supplementary file 1 [file ijms-23-14788-s001.zip › ijms-1969501-supplementary.pdf]

ARTICLE

Novel Dual Ache and ROCK2 Inhibitor Induces Neurogenesis Via PTEN/AKT Pathway in Alzheimer's Disease Model

Natália Chermont dos Santos Moreira[1], Elvira Regina Tamarozzi[2]; Jéssica Ellen Barbosa de Freitas Lima[1]; Larissa de Oliveira Piassi[1]; Ivone Carvalho[3]; Geraldo Aleixo Passos[1,4] and Elza Tiemi Sakamoto-Hojo[1,5*]

1 Department of Genetics, Ribeirão Preto Medical School, University of São Paulo—USP, Ribeirão Preto 14049-900, Brazil
2 Department of Biotechnology, School of Arts, Sciences and Humanities—USP, São Paulo 03828-000, Brazil
3 School of Pharmaceutical Sciences of Ribeirão Preto, University of São Paulo—USP, Ribeirão Preto 14040-900, Brazil
4 Laboratory of Genetics and Molecular Biology, Department of Basic and Oral Biology, School of Dentistry of Ribeirão Preto, University of São Paulo, Ribeirão Preto 14049-900, Brazil
5 Department of Biology, Faculty of Philosophy, Sciences and Letters at Ribeirão Preto, University of São Paulo—USP, Ribeirão Preto 14040-901, Brazil
*Correspondence: etshojo@usp.br; Tel.: +55-16-3315-3827

Supplementary data

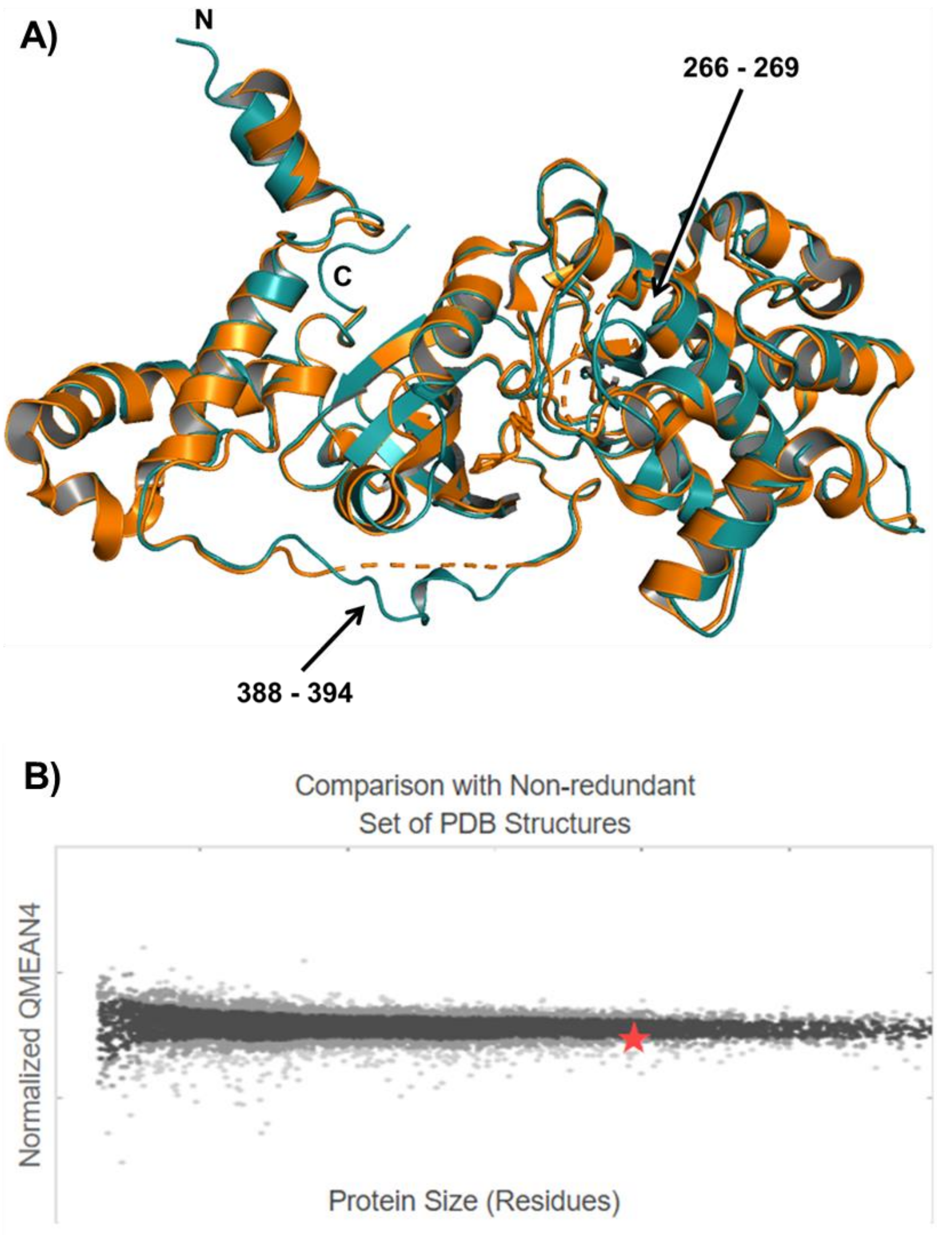

Figure S1. Structure of the complete ROCK2 protein. **(A)** Structure of ROCK2 protein (PDB ID: 6ED6 – Chain A) complete, with segments 266-269 and 388-394 modeled, shown in blue, aligned with the structure of ROCK2 with residues 266-269 and 388-394 still missing displayed in orange. (**B)** Results of the Z-score QMEAN to estimate the degree of confidence of the structural characteristics observed in the model in order to determine how close the structure is to its native folding. Z-score result, where the red X indicates the position of the evaluated structure in the same range of Z-score values as experimentally predicted structures.

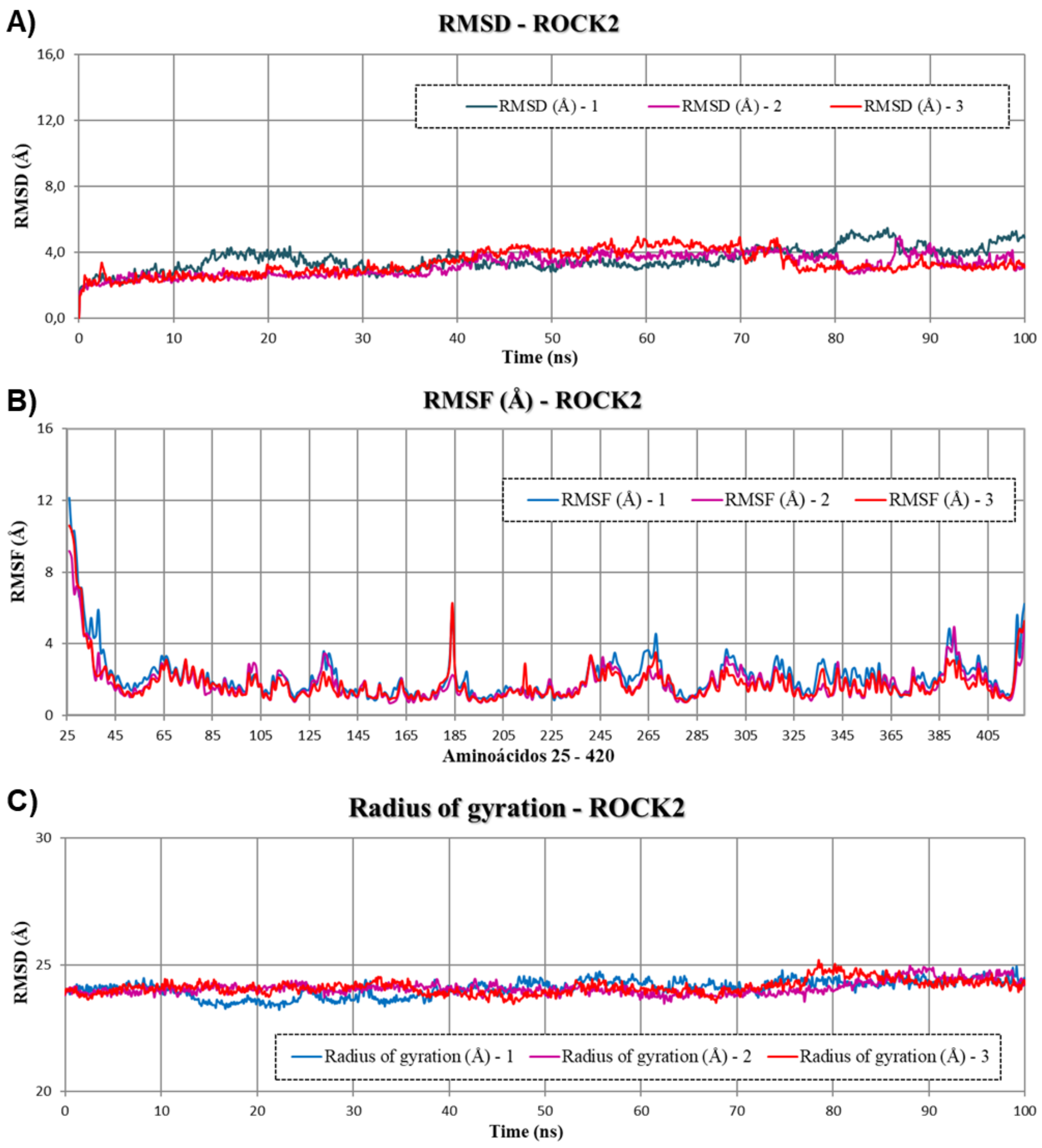

Figure S2. Trajectory of the DM simulation of the structure of ROCK2. **(A)** RMSD values of the coordinates of the Cα atoms as a function of the simulation time interval in triplicate of 100 ns. **(B)** Comparison of the structural variation per amino acid of the three simulations. RMSF values of the coordinates of the Cα atoms in relation to the initial structure. **(C)** Values for the structural compaction over time of the MD simulation in triplicate of 100 ns presented in the Rg graph.

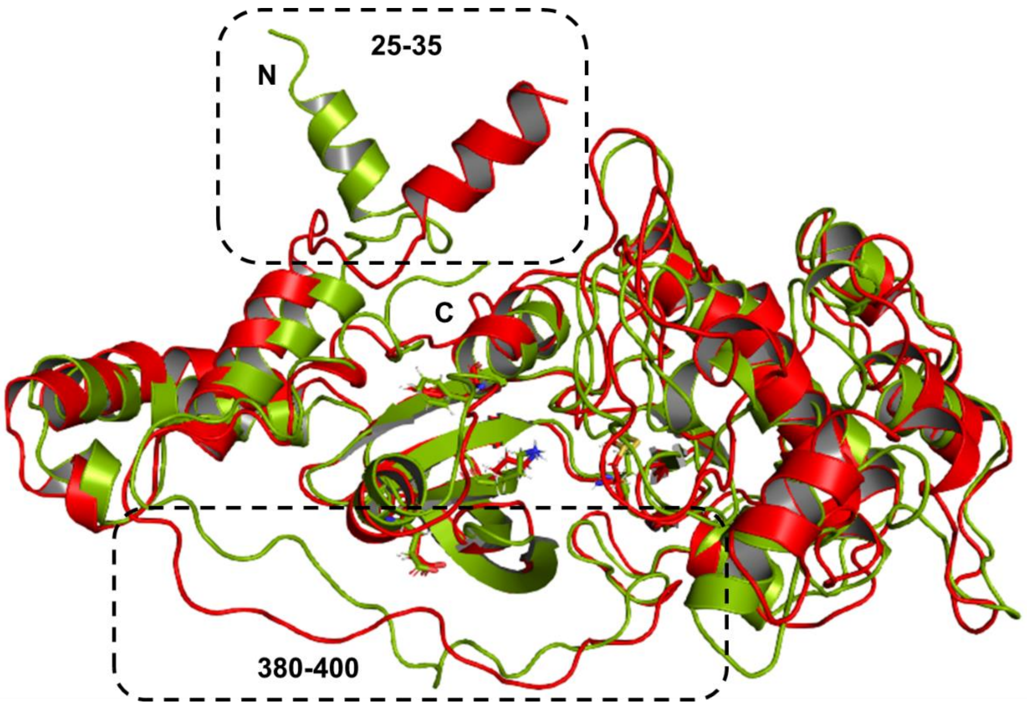

Figure S3. Alignment of ROCK2 structures at the start and end time of the DM simulation. Alignment of the initial structures at 0 ns (green) and final at 100 ns (red) of the DM simulation. The black dotted rectangles highlight the regions that have moved the most in relation to the structure's initial positioning.

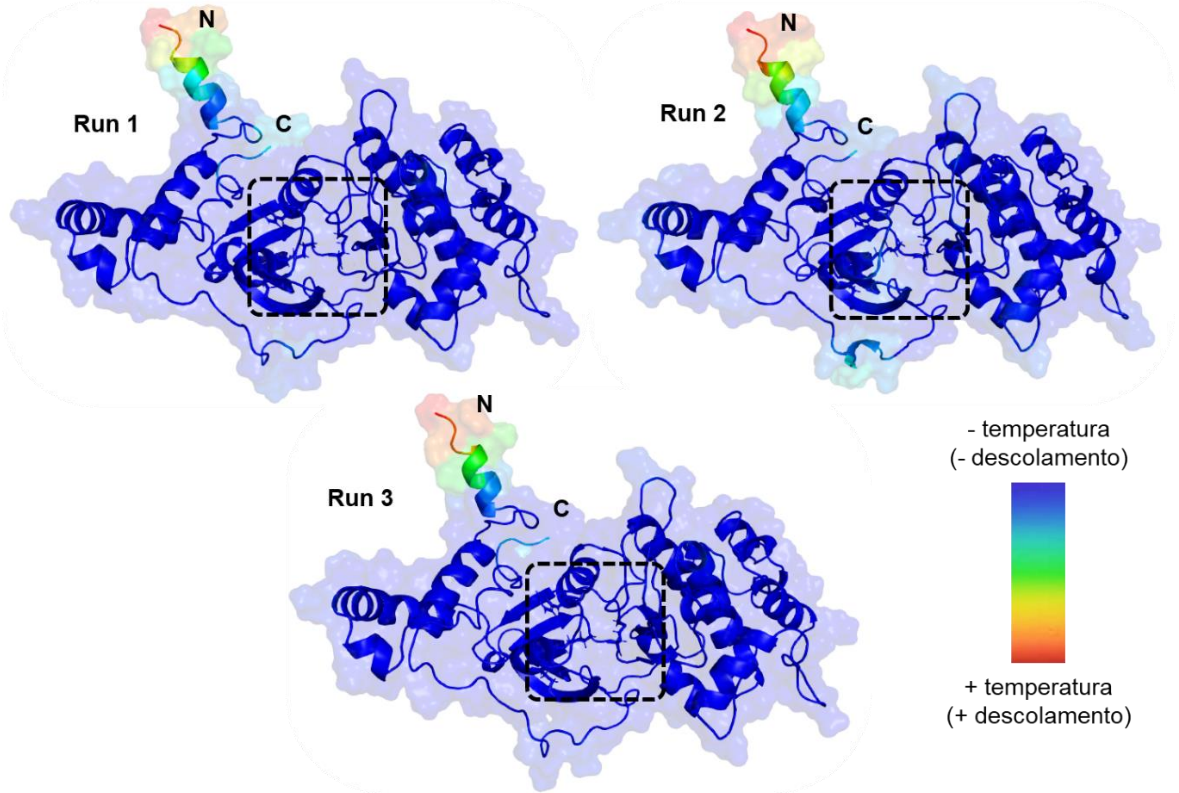

Figure S4. Structural behavior observed by the b-factor. Results of the triplicate MD simulation b-factors. The black dotted rectangle highlights the region of the binding site where the docking of the TA8amino compound will be directed.
